# A New Science for an Old(er) Population: Soviet Gerontology and Geriatrics in International Comparative Perspective

**DOI:** 10.1093/shm/hkac001

**Published:** 2022-02-14

**Authors:** Isaac McKean Scarborough

**Keywords:** geriatric medicine, gerontology, Soviet Union, Kiev, United Nations

## Abstract

Like most developed nations, the Soviet Union faced an unprecedented demographic shift during the latter half of the twentieth century, as its population aged and life expectancies grew significantly. Facing similar challenges as the USA or the UK, this article argues, the USSR reacted similarly and equally ad hoc, allowing biological gerontology and geriatrics to develop as sciences and medical specialisations with little central direction. When political attention was focused on ageing, moreover, the Soviet response remained largely comparable to the West’s, with geriatric medicine slowly overtaking research into the foundations of ageing and yet remaining sorely underfunded and underpromoted.

Demographically and scientifically, geriatrics (physicians’ medical practices of treating conditions common in later life) and biological gerontology (the study of the foundations of ageing) came of age with the USSR.^1^ At the time of the October Revolution in 1917, both disciplines existed largely in theory, and there was little of a worldwide aged population of which to speak. By the late 1950s, however, when the Soviet Union founded its own Institute of Gerontology in Kiev and began to invest in biological gerontology and geriatric medicine, worldwide demographics were changing. Increasingly large portions of the population in Europe, the USA, and the USSR alike were living into old(er) age, and there was increased focus and interest in both understanding the underpinnings of ageing and considering methods of treating its consequences.

While the USSR’s scientific and medical communities and practices have largely been considered at arm’s length to Western medicine and science—an isolated ‘other’ that operated separately from the West—the case of gerontology and geriatric medicine presents a different picture entirely.^2^ Especially in the post-war period and under the leadership of Stalin’s immediate successors, Nikita Khrushchev and Leonid Brezhnev, notwithstanding the Cold War between the USSR and the West, the disciplines developed largely in parallel across the Iron Curtain—and with important moments of cross-fertilisation.^3^ As in other medical disciplines, interactions between Soviet and Western scientists were often coloured by aspects of Cold War competition—but also developed on the basis of personal curiosity and scientific respect.^4^ Much as in the West, in the USSR gerontology and geriatric medicine were established by individual scientists and doctors, operating largely on individual initiative and pushing on their own for research and funding. While starting a few decades later than their colleagues in the UK or the USA, moreover, Soviet researchers and doctors quickly caught up in many regards in the 1960s and 1970s, conducting important research into the biological foundations of ageing and demonstrating valuable insights into the treatment and prevention of diseases common in old age. By the 1980s, their insights were being applied at the highest level at the UN, being taken into consideration by the US National Institute on Ageing (NIA), and laying the foundation for training programmes at the World Health Organization (WHO). In the field of gerontology and geriatrics, Soviet biomedical science was both well-connected and well-regarded internationally.

The field of Soviet gerontology and geriatrics also followed a similar *internal* trajectory to its Western counterparts. Throughout the twentieth century, there existed an international ambiguity in the field between studying ageing as a biological process and treating ageing as a field of medical intervention. Part of this uncertainty had to do with demographics: as this article will show, what was considered ‘older’ early in the twentieth century (sometimes as young as 40 years of age) was forced to adapt to rapidly expanding life expectancies, pushing definitions of ‘older persons’ generally past 60 and medical intervention and research alike in new directions.^5^ This changing field of study had notable impact on the discipline. As a group of gerontologists noted in the early 1990s, ‘There is little agreement as to what “gerontology” is, or, for that matter, what a “gerontologist” is’.^6^ In the beginning of the twentieth century, the answer seemed to be about the study of cellular growth, breakdown, and other biological aspects of ageing. As the decades passed, however, both the International Association of Gerontology (IAG) and individual national associations found their membership, and research base, shift towards the clinicians working with older persons. This was as equally true in the USSR as the USA or the UK, and in fact the USSR played an important role during the 1970s in terms of shifting the international discourse of gerontology and geriatrics firmly towards the latter.^7^ By tracking the institutional history of Soviet gerontology and geriatrics from the late 1950s to the late 1980s, this article places this history into its proper international context, suggesting important implications for the study of Soviet science and international medicine alike—normalising the former while complicating the latter.

## Founding the Institute of Gerontology: Three Stories

In early 1957, the story went, Nikita Khrushchev, First Secretary of the Communist Party of the Soviet Union (CPSU) and leader of the USSR, was visiting Bucharest, where he made a stop at the Romanian Institute of Geriatric Medicine named for the academician K. Parkhon. Impressed by the work done at the Institute, Khrushchev made a characteristic exclamation: ‘Why does the USSR not have this sort of institute?’ he demanded loudly. Pyotr Shelest, then the First Secretary of the Kiev City Party Committee, who had accompanied him to Bucharest, quickly spoke up. ‘Nikita Sergeevich’, he addressed Khrushchev, ‘We can open this sort of institute in Kiev!’ Khrushchev gave his approval, and within a year the Soviet Union’s very own Institute of Gerontology was opened in Kiev.^8^ Khrushchev later took advantage of the Institute’s research to undergo ‘anti-aging’ treatment in Kiev.^9^

Except that neither Khrushchev nor Shelest visited Bucharest in 1957, nor did Khrushchev demonstrate any particular interest in gerontology or geriatric medicine. Rumour aside, Khrushchev does not appear to have actually visited the Institute of Gerontology in Kiev or have received treatment there.^10^ There is another version the story, however, told by Dmitri Chebotaryov, who would go on to become the Director of the Soviet Institute of Gerontology. In December 1956, Chebotaryov later wrote in his memoirs, the Kiev-based correspondent of *Literaturnaia Gazeta* (the ‘*Literary Gazette*’, one of Moscow’s leading newspapers), Sviatoslav Ivanov, visited the Romanian Institute of Geriatric Medicine and published a glowing account of its work.^11^ Having read Ivanov’s article, the Minister of Healthcare of the USSR, Maria Kovrigina, directed the Ukrainian Ministry of Health to send a delegation to Bucharest and investigate the possibility of opening a similar institute. Pushed forward by the Minister’s initiative, Ukrainian ministry officials quickly laid the groundwork and the Soviet institute was opened shortly thereafter.^12^

Yet there were some issues with this story as well. A delegation was sent from the USSR to Bucharest to visit the Romanian Institute in January 1957, but it seemed unlikely that a single article, lodged on page two of a literary newspaper between a discussion about the quality of university education and an Australian travelogue would have so excited the Soviet Minister of Healthcare as to organise a delegation within weeks over the winter holidays.^13^ Instead, the archival record shows that the delegation was initiated not by Party figures or ministers, but by scientists and the directors of scientific institutes. On 20 November 1956—a full month before Ivanov’s article was published—the director of Moscow’s All-Union Institute of Experimental Endocrinology, Elena Vasiukova, wrote to Minister Kovrigina to inform her of the Romanian Institute’s success in treating ‘early ageing’ and requesting permission to send a ‘group of scientists including a clinical endocrinologist, a biochemist and a pharmacologist’ to visit the Institute.^14^ Kovrigina gave her approval, and on 15 January 1957, a group of four scientists arrived in Bucharest. In addition to Vasiukova (an endocrinologist), the delegation included Aleksander Vishnevskii, a pathologist and the Red Army’s Chief Surgeon, the biologist E.A. Kolli, and Dmitri Chebotaryov, a doctor, internist, and, at the time, the Chairman of the Ukrainian SSR’s Medical Council—essentially the state’s own ‘inside man’ in the group as the head of the Ukrainian branch of the Soviet Ministry of Healthcare’s ‘Fourth Main Directorate’ or medical service for Party leaders.^15^

Upon returning to Moscow and Kiev, the delegation filed a report to the Ministry of Healthcare, in which they commented on the work being done in Bucharest and recommended ‘the founding of a scientific-methodological centre’, dedicated to coordinating research related to ageing.^16^ The report left open what form this centre should take, or where it ought to be founded. Apropos of nothing, however, it mentioned that ‘In Ukraine after the death of professors Bogomolets and Nagornyi this work [gerontological research] has been left in the hands of individual doctors, and there is no systematic study of this problem’.^17^ This seemed to imply a potential location for the planned centre, as well as the real author of the report: Chebotaryov was the only Ukrainian in the delegation. In practice, though, matters were hardly as predetermined as later stories would imply. Upon receiving the delegation’s report, Kovrigina agreed in May 1957 to create a ‘Committee for gerontology and geriatrics’ under the Scientific Medical Council of the Ministry of Healthcare; this committee was then instructed to set the groundwork for an ‘All-Union Scientific-Research Institute for the Study of Longevity’, which was to be founded in 1958.^18^ Over the summer and fall of 1957 the committee discussed placing the planned institute in Moscow or Poltava and perhaps opening a filial in Tbilisi.^19^ When the Ministry finally settled on Kiev, this must have been something of a surprise. It was also almost certainly a product of background lobbying by Chebotaryov and Nikolai Gorev, a Kiev-based physiologist who had been included on the Committee on Gerontology and who would become the founding director of the Institute.

Thus when the Institute of Gerontology was formally opened in May 1958, it was the product of on-the-ground initiative, local lobbying, and the particular enthusiasm of scientists and medical administrators who saw an opportunity to promote both science and their own careers within the Soviet bureaucracy.^20^ The years 1956–57 were a particularly opportune moment in the biological sciences: Trofim Lysenko, who had previously exerted control over most of Soviet biology, had come under harsh and effective attack for his pseudo-scientific methods, and by 1956 had lost much of his authority.^21^ Especially in the context of Khrushchev’s attack on Stalin in his ‘secret speech’ to the 20th Congress of the CPSU in February 1956 and his broader campaign against the worst excesses of Stalinism, this meant that previously controversial topics, such as genetics and radiation therapy, were no longer matters of political concern; it also meant that Lysenko and his allies no longer controlled all of the purse strings and institutional stamps.^22^ It was, in other words, ‘The easiest time ever for one or another more or less prominent scientist to propose a big institute or laboratory’.^23^ For those who founded the Institute of Gerontology in Kiev, it was a particularly ripe moment to push for their own institution: 1956 was also the year of Soviet pension reform, when for the first time all urban workers were able to receive pensions upon retirement.^24^ This drove up public interest and publications on ageing and old age in general, and opened up space for administrators like Gorev and Chebotaryov to centralise Soviet gerontological research in a dedicated institute. Historical memory has largely removed this story of initiative and enthusiasm from the record, giving in to the assumed centralised nature of Soviet bureaucracy that colours even the accounts of those scientists and bureaucrats who demonstrated initiative in the first place. In fact, however, the Institute of Gerontology was founded in Kiev as the result of personal drive and dedicated lobbying—perhaps not that unlike the founding of similar institutes across the world.

## International Context

Before 1958 and the founding of the USSR’s Institute of Gerontology, there were very few full-time gerontologists and geriatric medicine specialists in the USSR. Much earlier in the late nineteenth and early twentieth century, in the years before the 1917 Bolshevik Revolution, Russia and Ukraine had produced world-famous gerontologists, but these individuals had tended to emigrate abroad. Ilya Mechnikov, the polymath biologist whose early twentieth-century works on cellular function laid the foundation for most of gerontology and immunology alike, was born outside of Kharkov but emigrated to Paris in 1888 and did his most important work abroad.^25^ In the early part of the twentieth century, Vladimir Korenchevskii, one of the few biologists working on cellular aging in Russia, also followed suit. In 1920, Korenchevskii left for London, where he worked in the Lister Centre for Preventative Medicine, and later at Oxford University.^26^

In the first decades of the USSR, research on the processes of ageing was limited but fruitful, with independent biomedical approaches developing under Aleksandr Nagornyi in Kharkov, Zakharii Frenkel’ in Leningrad and Aleksandr Bogomolets in Kiev.^27^ While Frenkel and Nagornyi conducted detailed research on the endocrinological and metabolic aspects of ageing, respectively, Bogomolets was largely unique in combining his scientific research on biological ageing with a growing national and international profile, in part by focusing on examples of exceptional old age. Bogomolets organised the first expeditions to study the ‘long-lived’ older persons (*dolgozhiteli*) of Abkhazia, organised the first conference on Ageing in the USSR (1938, Kiev), and published the first general-interest book on ageing in the country, entitled *Life Extension*, in 1940.^28^

By the mid-1950s, however, Soviet gerontology had reached a fallow moment: Bogomolets had died in 1946, and Nagornyi had followed in 1953 (Frenkel’ continued active research through the 1960s). Although their students continued their work in Kiev and Kharkov, the prominence of gerontology waned: as the Soviet Ministry of Healthcare report that recommended the foundation of an institute noted, there was currently no ‘systemic’ study of gerontology. For their part, Dmitri Chebotaryov and Nikolai Gorev, the Ukrainian tandem that brought about the founding of the institute in Kiev, were, respectively, an internist working on organ function during pregnancy and a pathophysiologist with expertise in blood flow. Both men had come into contact with Bogomolets and both had worked at the Physiological Institute that he founded in the 1930s.^29^ Neither, however, had worked on the medical or biological processes of ageing, nor considered themselves ‘gerontologists’. This title would come with their new positions at the head of the USSR’s first Institute of Gerontology.

This path was not, however, unusual for the time. Over much of the same period both the USA and the UK—two other countries to develop particularly expansive programmes of gerontological science and geriatric medicine in the twentieth century—also followed similar trajectories. In the USA, 1938 also marked the beginnings of gerontology’s formalisation with the publication of Edmund Cowdry’s *Problems of Ageing*, a large edited volume of material largely related to the biological processes of ageing.^30^ A cytologist from Washington University in St. Lewis, Cowdry brought together a large number of articles presented at a scientific conference held in June 1937 in Woods Hole, Massachusetts, which had been funded by the private Josiah Macy Jr Foundation.^31^ In 1940, Cowdry and others, together with financial backing from the same Foundation, convinced the US government to include a Unit on Ageing in the newly founded National Institutes of Health. As the Unit’s second director, Nathan Shock, later reported, this allowed for research to begin on the sociological and medical aspects of ageing: ‘A number of studies on age differences in physiological functions in humans were continued. Aging studies became a part of the ongoing research programs of the NIH’.^32^ During World War II, the American Geriatrics Society (1942) and Gerontological Society of America (GSA—1945) were founded; the latter organisation was largely run by Cowdry and Shock. Institutional growth was occurring, but as in the USSR, it remained in the domain of particular personalities.

In the UK, the situation was largely analogous. Having emigrated to London in 1920, Vladimir Korenchevsky worked independently for decades on research into cell function and hormones. In 1945, however, he was able to convince the recently founded Nuffield Foundation to fund a series of gerontological centres, including the Oxford Gerontological Research Unit, which Korenchevsky directed until 1951.^33^ Contemporaneously, geriatric medicine also became established with the founding of the British Geriatrics Society in 1947 and the licensing of ‘geriatric medicine’ as an officially recognised medical specialisation by the National Health Service (NHS) upon its founding in 1948. Largely working from the example of Marjory Warren, a doctor with the West Middlesex County Hospital, who had spent the 1930s and 1940s developing treatments for the chronic diseases experienced by many of her infirm and older patients, the first NHS consultant geriatricians began to work in the late 1940s and early 1950s in a number of hospitals.^34^ Basil William Sholto Mackenzie, 2nd Baron Amulree, for example, was appointed in 1949 head of the geriatric department at St. Pancras Hospital in London; at the time, this was the world’s only geriatric unit in a teaching hospital.^35^ A few years later in 1952, William Ferguson Anderson, a surgeon who would later became one of the UK’s leading specialists on geriatrics, was ‘appointed advisor in diseases of old age and chronic sickness to the then Western Regional Hospital Board (WRHB) in Scotland’.^36^ The ‘enthusiastic pioneers’ like Korenchevsky, Amulree and Anderson were in the process of laying the institutional groundwork for both biological gerontology and geriatric medicine, even as independent foundations and personal strength of will still underwrote many initiatives.^37^

In this context, when the Soviet Institute of Gerontology was founded in 1957–58 the Soviet Union was catching up to the level of institutionalisation previously obtained by American and British gerontology; the gap, however, was less than vast and quickly closed. When the Soviet endocrinologist Elena Vasiukova and immunologist P.D. Marchuk visited the Fourth Congress of the International Association of Gerontology in Merano and Venice, Italy, in June 1957, they found an event with ‘an overwhelming majority of papers given by Anglo-American scientists … everyone else was an absolute minority’.^38^ They were impressed by the size, with nearly 500 participants representing the 26 countries that were members of the IAG. The science, however, impressed them less: ‘Although questions of the biology of ageing were provided with a specialized congress section, the problem of ageing in a general biological light was not discussed’. Overall, they felt the work presented was often ‘circular’ (*eklipticheskii*) and unclear in its division between cause and effect.^39^ Seeing little reason why Soviet research into ageing, which she thought was at least the equal to its ‘bourgeoisie’ competition, should not receive the same attention, Vasiukova recommended ‘founding a scientific association of gerontologists and joining the international organization of gerontologists’, expanding gerontologists’ access to research funds and hospital patients, and holding a Union-wide conference on ageing in the USSR in 1958.^40^

## Catching up on Ageing

From this point, Soviet investment and organisation in gerontology and geriatrics began to catch up to its Western equivalents. The All-Union Scientific-Medical Association of Gerontologists and Geriatricians was founded in 1963, and quickly joined the IAG, while the Institute of Gerontology grew in Kiev. Aiming for a more ‘general’ approach to ageing that would combine clinical practice with fundamental research (and partially copying the structure of Nathan Shock’s Unit on Ageing in Baltimore, which worked closely with a local hospital), the Institute was founded with three interconnected divisions: experimental, clinical, and social gerontology. The idea from the beginning was to create a ‘streamlined structure that would allow for the actualization of complex and multidimensional experimental research in the field of gerontology’.^41^ Research at the Institute in the 1960s progressed in a number of fields: Chebotaryov, having become Director in 1961, oversaw the clinical wing; Vladimir Frol’kis, a leading Soviet specialist on the circulatory system, had been invited to join the Institute in 1958 and became head of its experimental wing in the early 1960s. Together with Nina Sachuk, the head of the Institute’s Demographics Unit, they developed research that attempted to bring together general theories of the physiological changes associated with ageing (Frol’kis’ realm), treatments for the ailments associated with these changes (Chebotaryov), and the statistical implications for the population at large (Sachuk).^42^ Throughout, there was an emphasis on linking fundamental research to clinical practice, using the hospital attached to the Institute as a ready base of research subjects from whom blood samples could be drawn, tests conducted upon, and experimental treatments applied (even if this might violate today’s assumptions about ethical guidelines.)^43^

Elsewhere in the Soviet Union, gerontological research and geriatric medicine also continued their development, if less synergistically. In Moscow, Leonid Komarov, a biological gerontologist, conducted early work on extending animal lifespans at the Institute of General Biology; in Leningrad, Vladimir Dil’man pursued work on the links between cancer, hormones, and cell growth at the Scientific-Research Cancer Research Institute named for N.N. Petrov; and back in Moscow, Zhores Medvedev worked largely in isolation at the Agricultural Academy named for Timeriazev on a new theory of ageing involving the accumulation of errors in the RNA production of proteins.^44^ At the same time, geriatric ‘cabinets’ and clinics specialising in geriatric medicine were being opened. Initially, these services were developed for the leaders of the Soviet government and CPSU, who by the early 1960s were no longer young men. Nikita Khrushchev, holding on as First Secretary of the Communist Party, had been born in 1894 and was already over 65; his to-be successor, Leonid Brezhnev, was a relatively spry 55 years old. As a 1959 letter to the Central Committee of the CPSU from the Soviet Ministry of Healthcare’s ‘Fourth Main Directorate’ described:

*The majority of the members (and candidate members) of the Presidium of the Central Committee of the CPSU, along with other Party leaders and state workers, are older than 50 years of age; some comrades are older than 60. Not all of them are entirely healthy, and many have various chronic diseases, including arteriosclerosis and hypertension; some have even had heart attacks.^45^*

These aging Party leaders, the report argued, needed ‘particular conditions in order to retain their capacity for work’ and should in all cases be provided with specialised medical services. By the early 1960s, moreover, the concern for the aged had moved beyond the highest tier of the Party to wider swaths of the Soviet population. The first ‘specialized clinic for the treatment of personal pensioners’ opened in Moscow in 1960 and quickly expanded its services to many groups of pensioners and veterans as Moscow’s 60th Clinical City Hospital.^46^ Outside of Moscow, the first ‘geriatric cabinets’ (*gerontologicheskie kabinety*) were opened in Kazan in 1957 and in the Gorky Oblast Clinical Hospital named for N.A. Semashko in 1961.^47^ By June 1962, there were at least 21 cabinets in operation across the USSR.^48^

As Soviet geriatric medicine and gerontological science expanded, it remained in contact with many international partners. At the Fifth Congress of the IAG, held in San Francisco, USA in August 1960, five Soviet participants offered papers, including Chebotaryov, Frol’kis and Medvedev.^49^ In the early 1960s, moreover, the Soviet doctor Mikhail Andreevich Akhmeteli, then working in the Division of Non-Communicable Diseases in the WHO’s European Regional Bureau in Copenhagen, arranged for a series of international meetings on gerontology to be held at the Soviet Institute of Gerontology in Kiev.^50^ The first WHO-sponsored seminar was held in May 1963 on the topic of ‘Protecting the Health of the Aged and Elderly People and Preventing Premature Ageing’. The WHO brought to Kiev leading experts in geriatric medicine from Eastern and Western Europe, including Vienna’s Dr Walter Doberauer (who had founded the Austrian Association of Geriatrics in 1955), the Netherlands’ Dr R.J. van Zonneveld, and leading experts from Bucharest, Prague and elsewhere.^51^ These and other medical professors spent a week discussing many aspects of geriatric medicine and gerontology, such as tests to determine whether a patient was experiencing ‘natural’ or ‘premature’ ageing, as well as ‘factors that are able to influence the speed of the ageing process’.^52^ These seminars were followed in 1965 and 1968 by month-long WHO-sponsored courses, in which some of the same Western experts, together with the world’s first university chair in geriatrics, Glasgow University’s William Ferguson Anderson, taught groups of Soviet and Eastern European doctors about advances in geriatric medicine.^53^

Across the USSR, moreover, biological gerontologists and geriatric medicine specialists alike were increasingly able to acquire foreign academic literature in their fields: although subscriptions to foreign journals were prohibitively expensive, most scientists received *Current Contacts*, a listing of researchers and article abstracts. This meant that they could send postcards asking for authors’ proofs to be sent directly to them; the majority of authors were happy to oblige.^54^ In Kiev, moreover, the Director of the Institute of Gerontology, Dmitry Chebotaryov, was particularly internationally active. He travelled to international conferences, including the Sixth, Seventh and Eighth Congresses of the IAG, became an advisor to the WHO, and for a brief moment, made the USSR the beating heart of worldwide gerontological and geriatric science in July 1972, when the Ninth Congress of the IAG was held in Kiev.^55^ In just under 15 years, the USSR had gone from a complete unknown to operating at ‘as high a level as anyone’ in the field of ageing.^56^

## Soviet Geriatrics on the International Stage

The decade after 1972 would prove the high point of Soviet influence in the field of gerontology and geriatrics, as well as a period of strong consolidation. Writing about the 1950s and 1960s in the USA, W. Andrew Achenbaum noted, ‘Gerontology emerged as a field of inquiry in an era of Big Science. As the scale of the scientific enterprise increased, researchers on aging defined and organized their work to take advantage of new opportunities. Investigations were undertaken by multidisciplinary teams’.^57^ Achenbaum might as well have been writing about *Soviet* gerontology in the 1970s. As the host institution for the Ninth Congress of the IAG, the Institute of Gerontology in Kiev was provided by the central Soviet government with millions of dollars of new equipment and resources unavailable to many other institutions. Access to foreign equipment and resources, such as the ever-necessary chemical reagents (*reaktivy*) that were needed in high quality and concentration to conduct biomedical research, also increased with the Institute’s links to the WHO and the United Nations.^58^ In 1969, Dr Tarek Shuman, the director of the recently founded UN Programme on Ageing, met Chebotaryov at the Eighth Congress of the IAG in Washington, DC. Shuman was ‘very impressed by the work of the Kiev Institute and invited Dr Chebotarev to visit the United Nations Headquarters in NY. During this visit, and in a letter of understanding between Dr Chebotarev and the UN, [they] established the basis of cooperation with the Kiev Institute in the field of aging’.^59^ As a result, Chebotaryov spent a decade (1972–83) as an advisor to the UN Programme on Ageing and UN Centre for Social Development and Humanitarian Affairs, which further increased his influence and control over scarce resources. While reflective of the same consolidating effects observed in the USA by Achenbaum and others, moreover, the Soviet Union’s pattern of centralising resources in ‘leading’ institutes was also heightened by the state’s close involvement in setting research priorities. The Soviet state, as represented by the Ministry of Healthcare or the Academy of Sciences, was the sole funder of research, meaning that it could effectively promote those institutes aligned with its ideological focus on synergy between biological gerontology and geriatric practice and side-line others. For non-Institute of Gerontology scientists, such as Lev Komarov in Moscow, this was an inequal struggle: funding dried up as research into ageing became centralised in Kiev.^60^ For those working at the Institute of Gerontology, however, such as Alexandre Sidorenko, who arrived as a young biologist in 1978, this consolidation meant that the Institute was now ‘one of the best academic institutions in the country’.^61^

By the early 1980s, Soviet gerontology and geriatric medicine were also being held in equally positive regard in many parts of the world. Having visited Kiev multiple times, William Ferguson Anderson was impressed by the ‘immense amounts of money..poured into’ Soviet research on ageing, as well as the emphasis in Kiev on diet and proper exercise.^62^ Ferguson’s student, John Brocklehurst, who would also later become one of Britain’s foremost geriatricians, also noted the Soviet Union’s ‘particular emphasis on physical culture’.^63^ Chebotaryov developed close and friendly relationships with Swiss, Austrian, Dutch and other European gerontologists and doctors, with whom he conducted a lively and cordial correspondence. Americans, such as Nathan Shock, who remained one of North America’s leading gerontologists, visited the Institute in Kiev and (if begrudgingly) noted the ‘substantial’ work being done in the Socialist bloc.^64^ Most institutions were less grudging in their respect. Dr Robert N. Butler, the founding director of the United States’ National Institute on Aging (NIA), which was formed in 1974, visited the Institute of Gerontology in Kiev at least twice (in 1978 and 1980); his local interlocutors reported that he was particularly interested in learning from the Institute’s model of integrated research and clinical work.^65^ The WHO continued to praise the ‘success’ of the Institute and develop closer formal ties with the Institute.^66^ On Chebotaryov’s initiative, the WHO formed an ‘Expert Committee on Planning and Organization of Geriatric Services’, which was meant to coordinate initiatives in the field of ageing. For its part, the UN Programme on Ageing made the USSR’s Institute of Gerontology and Chebotaryov a central element of its efforts to promote aging on the UN agenda. The head of the UN Programme, Dr Shuman, believed that ‘the Institute’s unique program, combining gerontology and geriatrics’ meant that ‘collaboration between the UN and the Soviet Union was always strong, particularly in the technical and professional areas’.^67^ With Shuman’s support, Chebotaryov hosted a planning meeting for a hoped-for World Assembly on Ageing during the Ninth Congress of the IAG in Kiev in 1972, and was a participant in preparatory discussions about the Assembly throughout the 1970s. At the final World Assembly on Ageing, held in Vienna in 1982, Chebotaryov acted as the country representative for Ukraine, while his then assistant director, Vladislav Bezrukov, was a deputy to the official chairman of the Congress, the American William Kerrigan.

The 1982 World Assembly on Ageing proved an important moment for Soviet gerontology and the international study of ageing alike. For the USSR, it demonstrated its status as an equal with previous leaders in the field, first and foremost the USA and the UK. While an American had been appointed chairman of the Assembly, this was understood by the UN and the USSR as a condition for American funding; Soviet experts had been just as involved as anyone else in the planning for the Assembly.^68^ Many of the provisions set forth in the Vienna International Plan of Action on Aging that was passed by the World Assembly, moreover, mirrored calls that the Institute in Kiev had been making for decades about the need to focus on diet, nutrition, preventative medicine and a holistic approach to the needs of older persons.^69^ What the World Assembly did not mention in any way, however, was gerontology as a biological science or the study of the fundamentals of aging. Instead, reflecting a broader international quantity–quality shift in understandings of gerontology, it held the study of aging as one primarily of amelioration, not of extension. The emphasis of earlier decades on understanding ageing and potentially expanding its scope (biological gerontology) had given way to making ageing, such as it was—and such as it had already lengthened demographically—increasingly pleasant and less marred by ailment (geriatric medicine).

The larger shift of funding, institutional support, and social emphasis towards geriatrics and clinical gerontology had been long in coming. In his memoirs, Nathan Shock argued that it was from 1972 on that ‘the IAG first showed signs of deviating from its original emphasis on science’, with ‘social and medical programs for the elderly … offered as an alternative approach’, but in fact tensions had existed from the founding of the organisation.^70^ Although many of the initial founders of the US Gerontological Society, such as Shock or Cowdry, or the IAG, such as Korenchevsky, were fundamentally research scientists and not clinicians, from the 1940s they faced the need to balance their interests against geriatric medicine, the foundations of which had been laid at around the same time.^71^ Just as in Kiev, where the Institute of Gerontology’s experimental wing competed for resources with the clinical division, so too did the IAG find itself of two minds. As early as the Second Congress of the IAG in St. Louis in 1951, moreover, there seemed a clear shift towards geriatrics: ‘many of the papers read at the congress dealt primarily with diseases of the elderly (diagnosis and treatment) and social problems (retirement, income maintenance, and social services). No clear distinction was made between the effects of aging and disease’.^72^ This movement towards social and medical programmes as the focus of aging research would continue over the coming decades across the world. By the 1980s in the USA, ‘social scientists and healthcare professionals, not bench scientists, now dominate[d] the GSA’.^73^ In the USSR, a similar shift occurred. While neither Leonid Brezhnev, Khrushchev’s successor at the head of the USSR from 1964 to1982, nor the Soviet Ministry of Healthcare paid much attention to gerontology, when they did, the focus was solidly on the ‘socio-medical problem of gerontology and geriatrics … and the training of doctors and medical personnel in gerontology and geriatrics’, as was outlined in a 1977 Order on medical services for older persons.^74^ For his part, Chebotaryov, a clinician and doctor by training, increasingly emphasised the Kiev Institute of Gerontology’s ‘geriatric hospital and an extensive rehabilitation program’.^75^ During his presidency of the IAG, Chebotaryov furthered this international trend, promoting institutional links with the UN and WHO, while providing noncommittal answers to biologists who wrote to him complaining that biological research was ‘sadly lacking as compared with rapidly developing research in the fields of geriatrics and social gerontology’.^76^

Doubtlessly, the shift towards an international institutional emphasis on ‘adding life to years, and not just years to life’ (as both Chebotaryov and the GSA liked to repeat)^77^ reflected a shift in personalities. Like Shock and Cowdry in the USA or Korenchevsky in the UK, the first director of the Institute of Gerontology in Kiev, Nikolai Gorev, had been an experimental scientist. In the late 1960s and 1970s, however, this early wave of ‘bench scientists’ was replaced with those, like Chebotaryov or Austria’s Doberauer, who tried to balance science and medicine, or those, like Anderson, Zonneveld, or the long-term director of the US NIA, Robert Butler, who were all clear advocates of geriatric medicine.^78^ At the same time, this shift also occurred in a context of undeniable demographic change. By the late 1970s, life expectancy at birth across Europe had reached 73 years for women and 68 for men; in the USA it was 78 and 70; and in the USSR—73 for women and 63 for men. This was a significant increase over the early 1940s, when the respective figures had been 65 (women) and 60 (men) in the USA and 50 and 44 in the USSR. European rates were highly varied in the years before World War II, but in the UK life expectancies at the time were 64 and 58; in France they were 61 and 54.^79^ Wherever one looked, more and more people were living into older age. As the UN resolved in the Vienna Plan of Action in 1982, moreover, this demographic shift towards an increasingly aged and aging society was not ‘an unexpected, unforeseeable event nor a random result of national and international development efforts. It is the first and most visible outcome of a sectorally-based approach to socio-economic development all over the world’. This outcome could be seen optimistically, as the UN tried to emphasise, pointing to the opportunity presented to guarantee that ‘the generally expanding lifespan of individuals the world over will be accompanied by efforts to fill these extra years with a sense of purpose and accomplishment’.^80^ Or, as the Russian philosopher Anatoly Baranov has suggested, one could read the numbers somewhat more pessimistically: having brought the majority of its population to the final boundary of its possible working life (60–70), there was little reason for industrial society to extend it further, and thus old age could be left as it was, with geriatric medicine to take the role of smoothing over the rough edges of chronic disease.^81^ Yet however interpreted, it was undeniable that by the late 1970s and early 1980s the population of the developed world was ageing; it is perhaps unsurprising in this context that geriatrics, as a medicine called upon to treat the diseases most common in old age, came to take precedence over the underlying science of aging itself.

## A Concluding Case Study: Geriatric Medical Specialists

**Fig. 1.**
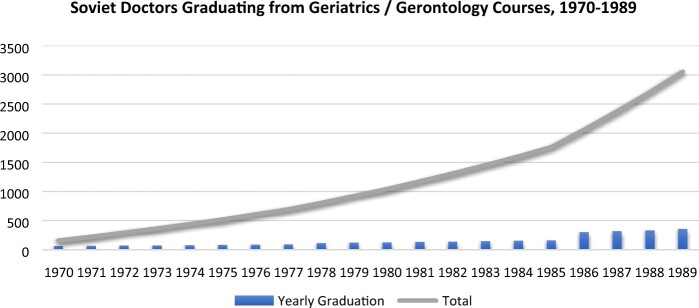
USSR Geriatric Medicine Training, 1970–1989 Calculated from: Chebotaryov, *Vospominaniia*, 16; GARF f. 8009, op. 50, d. 6550, l. 48; op. 51, d. 2404, ll. 17, 19 52; d. 4394, ll. 40, 42; d. 4395, l. 43.

One institutional response to the growth of aged populations was to train doctors in the treatment of diseases common in old age: to produce geriatricians or specialists in geriatric medicine. As noted above, the UK began this process much earlier than most other states, accepting ‘geriatric medicine’ as an official medical specialisation at the founding of the NHS in 1948 and opening the world’s first clinical departments of geriatric medicine at St. Pancras, London in 1949 and at Glasgow University in 1965. Other countries in Europe and elsewhere were somewhat slower to respond to the looming demographic shift, but responded in largely comparable ways. In the USSR, for example, the earlier WHO-sponsored training programmes at the Institute of Gerontology in Kiev were replaced in 1970 by the Soviet Union’s first Department of Gerontology and Geriatrics, which was opened in the Kiev Institute of Medical Post-Diploma Education.^82^ This department began to train doctors as geriatric specialists, providing them with a three-month course and practical work in the Institute’s network of geriatric clinics and homes for older persons. Each year, around 100 doctors received specialised training in this fashion, and in 1986 a second Department of Gerontology and Geriatrics was opened in the Leningrad Institute of Medical Post-Diploma Education, which began to train between 125 and 175 specialists in geriatric medicine each year.^83^ By 1989, more than 3,000 doctors had received this training at both Institutes (see Figure 1, below).

**Fig. 2.**
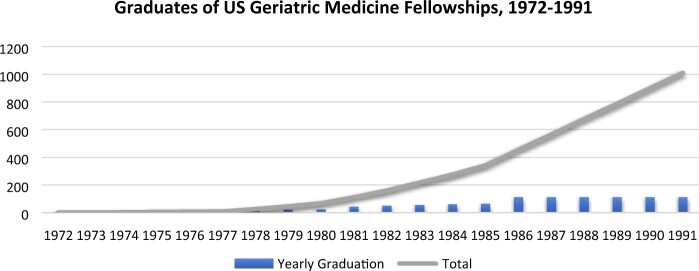
US Geriatric Medicine Fellowships, 1972–1991 Calculated from Leslie Libow, ‘A Geriatric Medical Residency Program: A Four-Year Experience’, *Annals of Internal Medicine*, 1976, 85, 641–47; Gregg A. Warshaw and Elizabeth J. Bragg, ‘The Training of Geriatricians in the United States: Three Decades of Progress’, *Journal of the American Geriatrics Society*, 2003, 51, 338–45; Leslie Libow, ‘The First Geriatric Residency – Fellowship in the United States’, *The Journals of Gerontology*, 2004, 59, 1165–66.

In the USA the development of geriatric medicine as a widespread specialisation occurred over a similar period and at similar rates.^84^ Although the American Geriatric Association had been founded in 1942, no formal Department of Geriatrics was created at any medical school before 1982, leaving the status of the specialisation somewhat unclear. In 1968 the American Medical Association formally approved ‘geriatrics’ as a specialisation, but only in 1972 was the first fellowship (postgraduate specialisation programme) in geriatric medicine created at the Mount Sinai School of Medicine in New York. Its founder, Dr Leslie Libow, would also go on to establish the second fellowship, at Long Island’s Jewish Institute for Geriatric Care. By the late 1970s, these fellowships were supplemented by a number of similar programmes funded by the NIA and the Veterans’ Health Administration (VHA) of the US Department of Veteran Affairs. The VHA was particularly influential and generous with its funding, and by the late 1980s there were 92 fellowships in geriatric medicine operating in the USA. Each fellowship programme trained one or two specialists a year; by 1991, approximately 1,000 specialists had received training in this fashion (see Figure 2, below).

These two graphs show notably similar trajectories in quantitative terms: while the USSR had produced three times as many geriatric medical specialists as the USA by 1989, there were also, in either absolute or per-capita terms, three times as many doctors.^85^ They also represent strikingly similar qualitative stories. In the USSR, strong personalities, such as Kiev’s Dmitri Chebotaryov, were at the centre of gerontology and geriatrics for decades, pushing for increased funding and training alike. When the first Department of Geriatrics opened in Kiev, for example, Chebotaryov served as its Chair. In the USA, Dr Libow remained instrumental to many efforts to promote geriatrics, helping to open the first Department of Geriatric Medicine at Mount Sinai in 1982. The NIA’s Robert Butler, an early advocate for older persons in US medicine, became the founding Chair of this department.^86^ The US government also became involved, initially through the promotion of the health of its veterans (through the VHA) and later through both the NIA and VHA hospitals. This mirrored the USSR’s efforts to promote the health of initially small (if also admittedly elite) cohorts, efforts that later bled into larger and wider programmes. In both countries, individual efforts were translated over time into institutional structures, much as earlier biological researchers, excited over the prospects of a new science for an older population, had driven the rise of gerontology in the 1940s and 1950s.

In both the USA and the USSR, moreover, government initiatives tended to follow on and back up individuals’ existing action, rather than enact entirely new programmes. In the USA, the founding of the National Institute of Aging and the funding it provided followed and essentially co-opted the work done by Butler, Libow, and others. In the Soviet Union, the Ministry of Healthcare’s decision to ‘organize in 1977–1978 in the form of an experiment consultative geriatric cabinets in Moscow and … certain large industrial centres’ was an experiment only in name: as this article has shown, these cabinets had been functioning for decades prior.^87^ It is also the case that even as government backing and scientific focus in both societies shifted from biological research into the foundations of aging to more disease-focused geriatric medicine, the overall importance of the field remained relatively limited. Although the founding of the National Institute of Ageing in 1975 had led to a significant increase in ageing-related funding, this remained paltry compared to other medical research: in 1976, the NIA received $19,288,000 in funding, compared to $761,727,000 provided to the National Cancer Institute and less than 1 per cent of the total NIH budget. (By 1991 the NIA budget had increased fifteen-fold to $323,752,000 but remained only 4 per cent of the NIH’s total budget of nearly $7.5 billion.)^88^ The provision of geriatricians was clearly below the needs of American hospitals and medical facilities, and medical students were hardly encouraged to pursue it as a specialisation. The geriatrician Louise Aronson, who attended Harvard Medical School in the late 1980s, has recalled learning ‘very little’ about geriatrics during her medical training and having it shunted aside in favour of more prestigious specialisations, such as surgery or internal medicine.^89^

In the Soviet Union, both gerontological research and geriatric medicine were equally low on the biomedical totem pole. With little application in the military or overlap with prestige sciences like physics or chemistry, gerontology lacked for funding; with a general focus on youth, paediatrics, and prevention in the Soviet medical system, geriatric medicine was also very poorly developed. (Mirroring Aronson’s comments, the Russian geriatrician Lidia Khoroshinina, who studied geriatric medicine in Leningrad in the late 1980s, noted that in the late Soviet period ‘there was essentially no support for gerontology’.^90^). As in the USA, priorities for medical research in the 1970s and 1980s included cancer, cardiology, paediatrics, and the like—never once was geriatrics listed as a ‘priority area’ (*osnovnoe napravlenie*) by the Ministry of Healthcare.^91^ As elsewhere in the European world and the USA, the focus in discussions about older persons was often on pensions and pension ages.^92^ Just as biological gerontology had been driven on both sides of the Iron Curtain by individual enthusiasts, so had it come to be eclipsed by geriatric medicine—and in turn, on both sides of the ideological divide, this medicine had grown in import while remaining largely secondary on the political radar. Neither in the USA nor the Soviet Union would it ever reach the importance of more targeted medical research or specialisations, a status it retains today, even as ageing populations in both societies grow in size and medical need.

In the final accounting the Soviet Union’s approach to gerontology and geriatric medicine proved anything but exceptional. Instead, it appeared to be confronting a global demographic trend in much the same way as its contemporaries: at first paying little attention, then allowing a biological and medical science to grow largely undirected and eventually putting some—if hardly sufficient—institutional and financial backing into the endeavours already begun by individual scientists and doctors. The historian Kate Brown, surveying the spatial and architectural similarities of Billings, Montana, and Karaganda, Kazakhstan, has suggested that ‘with the threat of the Cold War faded, there is more room to question whether knowledge itself has not been gridded into neat polarities, communist and democratic’.^93^ In the case of Soviet geriatric medicine and gerontology, this article would suggest, knowledge had indeed been gridded and artificially pulled about, thus obscuring factually similar and interlinked stories of scientific development and political inaction in the face of truly global and unavoidable demographic change.

## Footnotes

1 For the purposes of this article, a ‘gerontologist’ is understood as someone studying the biology of ageing, while ‘geriatrics’ refers to the medical practices of treating diseases commonly found amongst older populations, whether conducted by board-certified geriatricians or doctors or clinicians with less extensive training. While more dominant today, social gerontology was largely absent in the USSR.

2 This is partly due to a focus in many studies on the pre-war and Stalinist period, during which time divergences were more obvious. For important studies in this field, see Donald Filtzer, *The Hazards of Urban Life in Late Stalinist Russia: Health, Hygiene, and Living Standards* (Cambridge: Cambridge University Press, 2010); Susan Gross Solomon and John F. Hutchinson, eds, *Health and Society in Revolutionary Russia* (Bloomington: Indiana University Press, 1990); Frances Bernstein, Christopher Burton, and Dan Healy, eds, *Soviet Medicine: Culture, Practice, and Science* (Dekalb: Northern Illinois University Press, 2010); Paula A. Michaels, *Curative Powers: Medicine and Empire in Stalin’s Soviet Central Asia* (Pittsburgh: University of Pittsburgh Press, 2003); Benjamin Zajicek, ‘A Soviet System of Professions: Psychiatry, Professional Jurisdiction, and the Soviet Academy of Medical Sciences, 1932–1951’, in Susan Grant, ed., *Russian and Soviet Health Care from an International Perspective: Comparing Professions, Practice and Gender, 1880–**1960* (London: Palgrave, 2017), 97–117. During the Cold War, this emphasis was further coloured by the need to emphasise the faults of the Soviet medical system; see William A. Knuas, *Inside Russian Medicine: An American Doctor’s First-Hand Report* (New York: Everest, 1981); David D. Friedenberg, ‘Soviet Health Care System’, *Western Journal of Medicine*, 1987, 147, 214–17; Diane Rowland and Alexandre V. Telyukov, ‘Soviet Health Care From Two Perspectives’, *Health Affairs*, Fall 1991, 71–86.

3 For some important exceptions emphasising the international linkages of Soviet medicine, see Michael Z. David, ‘Vaccination against Tuberculosis with BCG: A Study of Innovation in Soviet Public Health, 1925–1941’, in Frances Bernstein, Christopher Burton, and Dan Healy, eds, *Soviet Medicine: Culture, Practice, and Science* (Dekalb: Northern Illinois University Press, 2010), 132–54; Paula A. Michaels, *Lamaze: An International History* (New York: Oxford University Press, 2014); Susan Grant, ‘Nurses Across Borders: Displaced Russian and Soviet Nurses after World War I and World War II’, *Nursing History Review*, 2014, 22, 13–36.

4 For an important emerging body of scholarship on cooperation and competition between Soviet and Western medical science during the Cold War, also see Nikolai Kremenstov, ‘In the Shadow of the Bomb: US-Soviet Biomedical Relations in the Early Cold War, 1944-1948’, *Journal of Cold War Studies*, 2007, 9, 41–67; Anna Geltzer, ‘In a Distorted Mirror: The Cold War and US-Soviet Biomedical Cooperation and (Mis)understanding, 1956–1977’, *Journal of Cold War Studies*, 2012, 14, 39–63; A.E. Birn and Nikolai Kremenstov, ‘“Socialising” Primary Care? The Soviet Union, the WHO and the 1978 Alma-Ata Conference’, *BMJ Global Health*, 2018, 3, 1–15; Dora Vargha, *Polio Across the Iron Curtain: Hungary’s Cold War with an Epidemic* (Cambridge: Cambridge University Press, 2018).

5 In the USSR, the term ‘older persons’ (*pozhilye liudi*) was defined from the early 1960s as those over 60, a category that would remain stable over the coming decades. See the report on the joint Soviet-WHO seminar ‘Okhrana zdorov’ia pozhilykh i starykh liudei i preduprezhdenie prezhdevremennogo stareniia’, Kiev, 14–22 May 1963, Tsentral’nii derzhavnii arkhiv vishchikh organiv vladi ta upravlinnia ukraini (henceforth TsDAVO), f. 4783, op. 1, d. 103, ll. 5-6. Notably, the benchmark of 60 was five years older than the standard age of women’s retirement in the USSR at 55. While Soviet scientists worked extensively on gender differences in ageing, social policy tended to flatten individuals into a masculine-model ‘norm’. On this point, see Danielle Levitt-Quist, ‘The Modern Babushka: Rethinking Older Women in Late Socialism’, in Susan Grant and Isaac Scarborough, eds, *Geriatrics and Ageing in the Soviet Union: Medical, Political, and Social Contexts* (London: Bloomsbury, 2022). For earlier and lower age ranges, also see Malford W. Thewlis, ‘History of the American Geriatrics Society’, *Journal of the American Geriatrics Society*, 1953, 1, 3.

6 Caroll L. Estes, Elizabeth A. Binney and Richard Culbertson, ‘The Gerontological Imagination’, *International Journal of Aging and Human Development*, 1992, 50, 50.

7 One of the few authors to consider the Soviet Institute of Gerontology and Soviet gerontology in general, Kavita Sivaramakrishnan, has suggested that the Soviet discipline remained focused on biological research even as Western emphasis shifted to social and medical concerns. Using a broader spectrum of Western and Soviet documentation, this article will suggest a much closer alignment and interchange between the two blocs. Cf. Kavita Sivaramakrishnan, *As the World Ages: Rethinking a Demographic Crisis* (Cambridge: Harvard University Press, 2018), 106–08.

8 This apocryphal story was related by Vladimir Anisimov, a leading Russian gerontologist, in St. Petersburg, Russia, March 2019.

9 As reported by Zhores Medvedev, *Soviet Science* (Oxford: Oxford University Press, 1978), 65.

10 Interview with the Director of the Institute of Gerontology, Vladislav Bezrukov, Kiev, September 2019.

11 Sv. Ivanov, ‘Starost’ mozhno lechit’’, *Literaturnaia gazeta*, 149, 15 December 1956, 2.

12 As per D.F. Chebotaryov, *Vospominaniia* (Kiev: IPTs ALKON NAN Ukrainy), 56–63.

13 When a delegation was sent to Romania and reported back, moreover, they went out of their way to criticise Ivanov’s articles as ‘misinformation’ (*dezorientiruiushchii*). See Gosudarstvennyi arkhiv Rossiskoi Federatsii (heneceforth GARF), f. 8009, op. 34, d. 370, l. 13.

14 E.A. Vasiukova Ministru zdravookhraneniia SSSR tov. M.D. Kovriginoi, 20.11.1956, GARF f. 8009, op. 34, d. 370, l. 22.

15 On the delegation’s makeup, see Otchet sovetskoi meditsinskoi delegatsii o poezdke v Rumynskuiu narodnuiu respubliku, g. Bukharest, GARF f. 8009, op. 34, d. 370, l. 1; on Chebotaryov and the Ministry of Healthcare’s ‘Fourth Main Directorate’ (*4-oe glavnoe upravlenie*), see Chebotaryov, *Vospominaniia*, 51–52; on the links between the Fourth Main Directorate (‘the important space where the most sacred secrets of the leaders of the country are kept’) and higher political echelons, see Evgenii Chazov, *Zdorov’e i vlast’: Vospominaniia ‘kremlovskogo vracha*’ (Moscow: Novosti, 1992), 17.

16 GARF f. 8009, op. 34, d. 370, l. 14.

17
*Ibid*.

18 Reshenie Kollegii Ministerstva zdravookhraneniia SSSR, Protokol no. 13 ot 16 maia 1957 g., ‘O meropriiatiiakh po probleme stareniia i dolgoletiia v SSSR’, GARF f. 8009, op. 2, d. 2347, ll. 14–15. In his memoirs, Chebotaryov claimed that this document included an order to open the Institute in Kiev, but in fact the word ‘Kiev’ did not appear anywhere therein; cf. Chebotaryov, *Vospominaniia*, 61.

19 GARF f. 8009, op. 2, d. 2347, ll. 18, 30–32.

20 On the formal founding of the Institute of Gerontology, see Chebotaryov, *Vospominaniia*, 62.

21 Lysenko’s destructive influence on Soviet genetics and biology as a result of his ‘Lamarkian’ agricultural projects and drive for power has been well documented; see esp. Nikolai Kremenstov, *Stalinist Science* (Princeton: Princeton University Press, 1997), 158–183; David Joravsky, *The Lysenko Affair* (Chicago: University of Chicago Press, 1986); Zhores Medvedev, *Vzlet i padenie Lysenko: Istoriia biologicheskoi diskussii v SSSR, 1929-1966* (Moscow: Kniga, 1993). In contrast to the frequently asserted argument that Lysenko remained dominant in most biological sciences after 1956, however, evidence collected by Loren Graham and Nikolai Dubinin has demonstrated his much-diminished role after the mid-1950s and his essential relegation to limited agricultural spheres in the decade before 1965. On this point, see Loren R. Graham, *Science, Philosophy, and Human Behavior in the Soviet* Union (New York: Columbia University Press, 1987), 140–42; N.P. Dubinin, *Vechnoe dvizhenie* (Moscow: Politizdat, 1973), 359–89, 401–02; as well as Dubinin’s May 1955 letter to Viacheslav Molotov and the Central Committee of the CPSU aggressively criticising Lysenko (Rossiskii gosudarstvennyi arkhiv noveishei istorii (henceforth RGANI) f. 5, op. 17, d. 516, ll. 3–7).

22 See the geneticist Nikolay Bochkov’s comments on Khrushchev and radiation research in the late 1950s (‘No problem for Khrushchev’) in his Interview with Peter Harper, Ufa, Russian Federation, 27 May 2005, transcript p. 5; on the wider impact of Khrushchev’s ‘secret speech’ see Susanne Schattenberg, ‘“Democracy” or “despotism”? How the Secret Speech was translated into everyday life’, in Polly Jones, ed., *The Dilemmas of De-Stalinization: Negotiating cultural and social change in the Khrushchev era* (London: Routledge, 2006), 64–79.

23 Medvedev, *Soviet Science*, 64.

24 On the 1956 pension reform, see Lukas Mucke, *Die allgemeine Altersrentenversorgung in der UdSSSR* (Stuttgart: Franz Steiner Verlag, 2013); Alissa Klots and Maria Romasheva, ‘The Right to a Permanent Collection: Archiving the Lives of Soviet Pensioners’, in Susan Grant and Isaac Scarborough, eds, *Geriatrics and Ageing in the Soviet Union: Medical, Political, and Social Contexts* (London: Bloomsbury, 2022).

25 In Western sources, Mechnikov is frequently considered a ‘European’ scientist and is known by the Francophone spelling of his name, Elie Metchnikoff. See W. Andrew Achenbaum, *Crossing Frontiers: Gerontology Emerges as a Science* (New York: Cambridge University Press, 1995), 23–33. For his importance to Soviet gerontology, see A.A. Bogomolets, *Prodlenie zhizni* (Kiev: Izdatel’stvo Akademii nauk USSR, 1940), 27–33; Chebotaryov, *Vospominaniia*, 54.

26 Nathan W. Shock with George T. Baker III, *The International Association of Gerontology: A Chronicle, 1950–**1986* (New York: Springer, 1988), 6–7.

27 On Nagornyi, see V.V. Bezrukov and Iu.K. Duplenko, ‘The Legend of Gilgamesh: Attempts at its Realization in Soviet Gerontology’, in Susan Grant and Isaac Scarborough, eds, *Geriatrics and Ageing in the Soviet Union: Medical, Political, and Social Contexts* (London: Bloomsbury, 2022); on Frenkel’ – V. N. Anisimov, *Gody priveredlivye* (St. Petersburg: Eskulap, 2014), 186–87; Z.G. Frenkel’, *Udlinenie zhizni i deiatel’naia starost’* (Moscow: Akademiia meditsinskikh nauk SSSR, 1949). For an overview of pre-1930s Soviet experimental biology and its relation to gerontology, see Nikolai Kremenstov, *Revolutionary Experiments: The Quest for Immortality in Bolshevik Science and Fiction* (Oxford: Oxford University Press, 2014). For some concrete examples from this period, see A.S. Dogel’, *Starost’ i smert’* (Petrograd: Mysl’, 1922); E.S. London and I.I. Kryzhanovskii, *Bor’ba za dolgovechnost’* (Petrograd: Put’ k znaniiu, 1924). I am grateful to Mariya Tutorskaya of the Russian Medical Museum for bringing these latter sources to my attention.

28 On Bogomolets and his work, see Chebotaryov, *Vospominaniia*, 45–60; Bogomolets, *Prodlenie zhizni*. Bogomolets’ book was also translated and published in the USA: Alexander A. Bogomolets, *The Prolongation of Life*, trans. Peter V. Karpovich and Sonia Bleeker (New York: Duell, Sloan and Bleeker, 1946).

29 Gorev was working at the Institute of Physiology named for A.A. Bogomolets in 1957 (GARF f. 8009, op. 2, d. 2347, l. 6), while Chebotaryov had worked there from 1945 to 1954 (*Vospominaniia*, 45–48).

30 Edmund Vincent Cowdry, ed., *Problems of Aging* (London: Bailliere, Tindall and Cox, 1939); this publication was also paid for by the Josiah Macy Jr. Foundation; see ‘Books received: Problems of Ageing’, *Nature*, 1939, 144, 995–996.

31 Hyung Wook Park, ‘Edmund Vincent Cowdry and the Making of Gerontology as a Multidisciplinary Scientific Field in the United States’, *Journal of the History of Biology*, 2008, 41, 529–72.

32 Shock with Baker, *The International Association*, 4–5.

33
*Ibid*., 7; Chebotaryov, *Vospominaniia*, 55.

34 John Grimley Evans, ‘Geriatric medicine: a brief history’, *BMJ*, 1997, 315, 1075–76.

35 Michael J. Denham, ‘Lord Amulree (1900-83): The Indefatigable Advocate of Older Persons’, *Journal of Medical Biography*, 2006, 14, 236.

36 W.F. Anderson, *A Fine Fellow, But … The Memoirs of Professor Sir William Ferguson Anderson, 1914–**2001* (Cambridge: Self Published, 2007), 110.

37 Quote from John C. Brocklehurst, ‘Geriatric Medicine in Britain – the Growth of a Speciality’, *Age and Ageing*, 1997, 26, 5. For a rich history of the development of British geriatric medicine, see Pat Thane, *Old Age in English History: Past Experiences, Present Issues* (Oxford: Oxford University Press, 2020), 436–58.

38 Otchet o poezdke sovetskoi delegatsii na Mezhdunarodnyi kongress po gerontologii, GARF f. 8009, op. 34, d. 319, l. 12. Vasiukova’s evaluation was shared by Nathan Shock, who wrote that, to judge by the 1957 Congress, ‘support of gerontology and geriatrics was largely an Anglo-American affair’. See Shock with Baker, *The International Association, 47.*

39 GARF f. 8009, op. 34, d. 319, ll. 2, 4.

40 GARF f. 8009, op. 34, d. 319, ll. 12–13. It is worth noting that this was the first IAG Congress that had been attended by Soviet scientists. The IAG claimed that there had been 2 Soviet representatives at the 2nd Congress in St. Louis in 1951 (Shock with Baker, *The International Association*, 22), but these were former Soviet citizens who had emigrated to the US and were studying in St. Louis (see ‘Index of participants’, *Journal of Gerontology* 1951, 6, supp. 3, 187, 198-A).

41 Chebotaryov, *Vospominaniia*, 64.

42 On Frol’kis, see V.V. Bezrukov and Iu. K. Duplenko, ‘Vydaiushchiisia ukrainskii fiziolog i gerontolog V.V. Frol’kis’, *Uspekhi gerontologii* 2000, 13; Iu. K. Duplenko, *Starenie: ocherki razvitiia problem* (Leningrad: Nauka, 1985), 113; on Sachuk, Chebotaryov, *Vospominaniia*, 70; for some comment on both, Shock with Baker, *The International Association*, 95.

43 Interview with Vladislav Bezrukov, Director of the Institute of Gerontology, Kiev, September 2019.

44 On Komarov, see T.A. Belova and S.E. Borisov, ‘Lev Vladimirovich Komarov – Uchenyi-gerontolog i obshchestvennyi deiatel’’, *Vestnik gerontologicheskogo obshchestva Rossiiskoi akademii nauk* 11-12, 2000; on Dil’man – Anisimov, *Gody priveredlivye*, 49; on Medvedev in the early 1960s – Zhores Medvedev, *Opasnaia professiia* (Moscow: Vremia, 2019), 68.

45 Pis’mo v TsK KPSS no. 12241 ot 09.04.1959, RGANI f. 3, op. 29, d. 27, ll. 33–34.

46 On the 60th Hospital’s beginnings, see RGANI f. 3, op. 29, d. 27, l. 129; on its closure in January 2019, see Nina Davletzianova, ‘“Khoziaeva tak ne delaiut”. Zakryta edinstvennaia v Rossii bol’nitsa dlia pozhilykh’, Radio Svoboda, 03 February 2019; online: https://www.svoboda.org/a/29748828.html; accessed 09 January 2022.

47 On Kazan, see TsDAVO, f. 4783, op. 1, d. 27, l. 41, citation courtesy of Susan Grant; on Gorky (Nizhnyi Novgorod), see Boris Anatol’evich Tolchenov, ‘Zdorov’e pozhilykh i problem organizatsii territorial’noi gerontologicheskoi sluzhby’ (Diss. na soiskanie uchenie stepeni kandidata meditsinskikh nauk, Moskovskaia meditsinskaia akademiia im. Sechenova, 1996), 32.

48 TsDAVO f. 4783, op. 1, d. 72, l. 57.

49 Shock with Baker, *The International Association*, 68, 74–75.

50 Dr Akhmeteli was instrumental in organising the seminar and many links to European organisations. See Anderson, *A Fine Fellow*, 128; TsDAVO f. 4783, op. 1, d. 103, l. 19ob-20; d. 105, l. 105. After a stint in the Soviet Ministry of Healthcare’s Division for Foreign Relations (*Upravlenie vneshnikh snoshenii*), Dr Akhmeteli would also return from 1971 to 1977 as Head of the Division for non-communicable diseases in the WHO Geneva Headquarters. See TsDAVO f. 4783, op. 1, d. 146, l. 1; ‘Mikhail Andreevich Akhmeteli’, WHO Employment Record, WHO Archive, Geneva.

51 On Doberauer and his 1960s work, see Martina Steinhardt, ‘Obituary: Walter Doberauer, 1912-1989’, *Experimental Gerontology*, 1990, 25, 3–5; for Zonnevald’s contemporaneous work, see R.J. van Zonneveld, ‘Social Geriatrics’, *Gerontologia clinica*, 1962, 4, 87–93.

52 Otchet o Seminare, sozvannom Vsemirnoi organizatsiei zdravookhraneniia. Kiev, 14–22 maia 1963 goda, TsDAVO f. 4783, op. 1, d. 103, l. 11. List of foreign participants on ll. 18–19. Also see Chebotaryov, *Vospominaniia*, 145–46.

53 Anderson, *A Fine Fellow*, 128; Chebotaryov, *Vospominaniia*, 146; TsDAVO f. 4783, op. 1, d. 146, l. 3–13; 51–54; 91.

54 Author’s interview with Dr Alexandre Sidorenko, Vienna, Austria (via Skype), August 2019. Also see Medvedev, *Soviet Science*, 63–64 on the increasing availability of foreign academic publications.

55 As the largest event hosted by the Institute of Gerontology, the Ninth Congress of the IAG features prominently in many accounts; for one, see Chebotaryov, *Vospominaniia*, 97–118.

56 Interview with Dr Sidorenko.

57 Achenbaum, *Crossing Frontiers*, 121.

58 On the need for and acquisition of foreign chemical reagents, see Medvedev, *Soviet Science*, 114–15; also Chebotaryov’s correspondence with Lester J. Perlman of the Morton Chemical Company (Illinois, USA) from 30 July 1971, TsDAVO f. 4783, op. 1, d. 322, l. 1.

59 Author’s personal correspondence with Dr Tarek Shuman (email), letter of 01 August 2019.

60 Author’s personal correspondence with Dr Igor Vladimirovich Vishev (email), letter of 01 April 2019. Others, such as the gerontologist Zhores Medvedev, faced much worse consequences, including forced psychiatric treatment and ultimately exile, albeit not strictly as a result of his gerontological research. On Medvedev’s experience, see Medvedev, *Opasnaia professiia*.

61 Interview with Sidorenko.

62 Anderson, *A Fine Fellow*, 128–30.

63 J.C. Brocklehurst, ‘Introduction: The Development of Geriatric Care in Advanced Societies’, in J.C. Brocklehurst, ed., *Geriatric Care in Advanced Societies* (Lancaster: MTP, 1975), 3.

64 Shock with Baker, *The International Association*, 164.

65 On Butler and the founding of the NIA, see Achenbaum, *Crossing Borders*, 215–16; Claude Denson Pepper with Hays Gorey, *Pepper: Eyewitness to a Century* (San Diego: Harcourt, 1987), 257. On his trips to Kiev, see Chebotaryov, *Vospominaniia*, 81; interview with Bezrukov.

66 See, for example, TsDAVO f. 4783, op. 1, d. 105, l. 11.

67 Author’s personal correspondence with Dr Tarek Shuman (email), letter of 06 September 2019.

68 Author’s personal correspondence with Dr Tarek Shuman (email), letter of 01 August 2019.

69 United Nations, *Vienna International Plan of Action on Aging* (UN: New York, 1983).

70 Shock with Baker, *The International Association*, 153.

71 For more on these foundations, see John E. Morley, ‘A Brief History of Geriatrics’, *Journal of Gerontology: Medical Sciences*, 2004, 59A, 1134.

72 Shock with Baker, *The International Association*, 23.

73 Achenbaum, *Crossing Frontiers*, 134.

74 Prikaz Ministerstva zdravookhraneniia SSSR no. 347, 19.04.1977, ‘O meropriiatiiakh po organizatsii meditsinskogo obespecheniia lits starshikh vozrastov’, GARF f. 8009, op. 50, d. 6272, ll. 281–86.

75 Shock with Baker, *The International Association*, 95.

76 From Chebotaryov’s letter to Prof. Carl Eisdorfer on 14 March 1973 in response to letters received from Profs. Verzar and Hall, TsDAVO f. 4783, op. 1, d. 483, l. 15.

77 This was in fact the GSA motto from 1940. See ‘The fountain of youth’, *Journal of Gerontology*, 1, 1946; Chebotaryov, *Vospominaniia*, 94. The phrase was also later popularised in the UK by Basil William Sholto Mackenzie, 2nd Baron Almuree. See Lord Amulree, *Adding Life to Years* (London: National Council of Social Service, 1951).

78 Butler had made this clear before his appointment as head of the NIA; see Robert N. Butler, *Why Survive? Being Old in America* (Harper: New York, 1975); also Interview with Robert N. Butler, Atlantic Philanthropies Oral History Project, 10 April 2006, Columbia University, transcript pp. 5–6.

79 For the pan-European figures, see Brocklehurst, ‘Introduction’, 2; for the USA, UK and French figures, see the data available through Human Mortality Database at https://www.mortality.org/. For the USSR, see Michael Ryan, ‘Life Expectancy and Mortality Data from the Soviet Union’, *British Medical Journal*, 1988, 296, 1513. One reason for the lag in male life expectancy in the USSR was high rates of alcohol consumption; see Mark Lawrence Schrad, *Vodka Politics: Alcohol, Autocracy, and the Secret History of the Russian State* (Oxford: Oxford University Press, 2014), 275–77.

80 United Nations, *Vienna International Plan*, 15–16.

81 A. Baranov, ‘V god smerti moego pokoleniia’, in M.B. Khodorkovskii, ed, *Postchelovechestvo* (Moscow: Algoritm, 2006), 270–301.

82 Chebotaryov, *Vospominaniia*, 16.

83 See Otchet Kievskogo gosudarstvennogo instituta usovershenstvovaniia vrachei MZ SSSR za 1977 god, GARF f. 8009, op. 50, d. 6550, l. 48; Uchebno-proizvodstvennyi plan povysheniia kvalifikatsii vrachei po Leningradskomu GIDUVu, GARF f. 8009, op. 51, d. 2404, l. 52; Otchet o rabote GIDUVa im. S.M. Kirova z 1989 god, GARF f. 8009, op. 51, d. 4395, l. 43.

84 Not only were the US and USSR continuously comparing each other during the Cold War, the similar population size and trajectory of development in geriatrics make the USA a better point of comparison for the USSR in terms of geriatric medical training. Figures for the UK may be similar, but on a different timeframe and scale, making direct comparison difficult at best.

85 In 1989, there were 1,278,000 Soviet physicians, or approximately 44.5 per 10,000 of the population; in the USA, the latter figure was 18 per 10,000 or less than 450,000 physicians in total. For Soviet statistics, see Ellen K. Cromley and Peter R. Craumer, ‘Regional Patterns of Medical Availability in the Former Soviet Union’, *Post-Soviet Geography*, 1992, 33, 204. For the US, see the World Bank’s dataset ‘Physicians (per 1,000 people) – United States’. Online: https://data.worldbank.org/indicator/SH.MED.PHYS.ZS?locations=US; accessed 7 October 2021.

86 See Interview with Robert N. Butler, transcript p. 6.

87 Prikaz Ministerstva zdravookhraneniia SSSR no. 347, 19.04.1977, ‘O meropriiatiiakh po organizatsii meditsinskogo obespecheniia lits starshikh vozrastov’, GARF f. 8009, op. 50, d. 6272, ll. 281–86.

88 As calculated from the historical appropriations figures provided by the National Institutes of Health; see NIH Almanac, ‘Appropriations (Section 1)’. Online: https://www.nih.gov/about-nih/what-we-do/nih-almanac/appropriations-section-1; accessed 09 January 2022.

89 Louise Aronson, *Elderhood: Redefining Aging, Transforming Medicine, Reimagining Life* (New York: Bloomsbury, 2019), 67.

90 Interview with Lidia Pavlovna Khoroshinina, St. Petersburg, March 2019.

91 See, for example, Proekt postanovleniia, ‘Ob osnovnykh napravleniiakh razvitiia zdravookhraneniia i okhrany zdorov’ia naroda v strane v dvenadtsatoi piatiletke i do 2000 goda’, (1986), GARF f. 8009, op. 51, d. 2171, l. 296; for earlier years, see a similar project passed in 1968: GARF, f. 8009, op 50, d. 502.

92 On Soviet pensions and the treatment of older persons, see Mucke, *Die allgemeine Altersrentenversorgung*; Stephen Lovell, ‘Soviet Socialism and the Construction of Old Age’, *Jahrbucher fur Geschichte Osteuropas*, 51, 2003, 564–85. For the broader importance of pensions as a sign of government engagement with older persons across the Iron Curtain, see James Chappel, ‘Old Volk: Aging in 1950s Germany, East and West’, *The Journal of Modern History*, 2018, 90, 792–833; Sivaramakrishnan, *As the World Ages*, 108.

93 Kate Brown, ‘Gridded Lives: Why Kazakhstan and Montana are Nearly the Same Place’, *American Historical Review*, 2001, 106, 21. There is an important body of literature that has begun to suggest the ‘normalcy’ of Soviet practice in many different aspects of its economic and social policy. See, for example, Stephen Kotkin, ‘Modern Times: The Soviet Union and the Interwar Conjecture’, *Kritika* 2001, 2, 111–64; Oscar Sanchez-Sibony, ‘Capitalism’s Fellow Traveler: The Soviet Union, Bretton Woods, and the Cold War, 1944-1958’, *Comparative Studies in Society and History*, 2014, 56, 290–314; Michael David-Fox, *Crossing Borders: Modernity, Ideology, and Culture in Russia and the Soviet Union* (Pittsburgh: University of Pittsburgh Press, 2017).

